# Peer-led safer supply and opioid agonist treatment medication distribution: a case study from rural British Columbia

**DOI:** 10.1186/s12954-023-00883-x

**Published:** 2023-10-25

**Authors:** Marnie Scow, Jenny McDougall, Amanda Slaunwhite, Heather Palis

**Affiliations:** 1 BC Centre for Disease Control, University of British Columbia, 655 W 12th Ave, Vancouver, BC V5Z 4R4 Canada; 2 School of Population and Public Health, University of British Columbia, 2206 E Mall, Vancouver, BC V6T 1Z3 Canada; 3 Coalition of Substance Users of the North, Northern British Columbia, Quesnel, BC Canada; 4 Department of Psychiatry, University of British Columbia, 2255 Wesbrook Mall, Vancouver, BC V6T 2A1 Canada

**Keywords:** Peer-led, Harm reduction, Medication distribution, Opioid agonist treatment, Safe supply

## Abstract

**Background:**

British Columbia (BC) has been facing a public health emergency of overdose since 2016, with rural regions of the province facing the highest rates of death. Peers (in this case, people with lived experience of substance use) are known to be effective patient navigators in health systems and can play a role in connecting patients to care and reducing overdose risk.

**Case presentation:**

We outline a peer-led program focused on opioid agonist treatment and prescribed safe supply medication delivery that began in March 2020 at a clinic in rural BC. The peer takes an Indigenous harm reduction approach and is focused on meeting the needs of the whole person. The peer has regular contact with approximately 50 clients and navigates medication delivery and appointments for approximately 10–15 people each day. Clients have been retained on the medication, and experienced improvement in other outcomes, including securing housing, employment and managing acute and chronic health conditions. The peer has established contact with clients since March 2020 to support engagement with health care and continuity of medication access. This program highlights the importance and value of peer-led work and need for further investments in peer-led programs to respond to the unregulated drug poisoning crisis.

**Conclusions:**

This peer-led intervention is a promising approach to engaging people who remain disconnected from health services in care in a rural community. This model could be adapted to other settings to support patient contact with the health system and medication access and continuity, with the ultimate goal of reducing overdose risk.

## Background

Unregulated drug poisoning (i.e. overdose) is a public health emergency, with overdose death rates continuing to rise in the context of COVID-19 across North America [1–3]. British Columbia (BC) has been facing a public health emergency of overdose since 2016, with overdose rates consistently doubling the national average [4]. In BC, there were more than 1200 overdose deaths in the first six months of 2023, and overdose remains the leading cause of death for British Columbians aged 10–59, now accounting for more deaths than homicides, suicides, accidents, and natural diseases combined [4]. A number of novel interventions have been introduced to curb overdose deaths in BC, including expanded opioid agonist treatment (OAT) options, and a prescribed safer supply policy that provided guidance for physicians and nurse practitioners to prescribe alternatives (i.e. opioids, stimulants, benzodiazepines, alcohol withdrawal management medications) to the unregulated drug supply [5, 6].

Nevertheless, rates of overdose deaths have continued to rise in BC and have consistently disproportionately affected Indigenous peoples. For example, First Nations people are overrepresented in toxic drug poisoning deaths, making up just 3.2% of the province’s population, but 15.3% of toxic drug poisoning deaths in 2022 [7]. This is particularly true in Northern BC, where rates of overdose death are the highest in the province [4]. Many First Nations communities in BC are located in rural and remote locations, where access to health services may be met with a number of challenges including traveling long distances [7]. People who use drugs in rural and remote communities face increased barriers to services, including limited primary care access and long travel distances required to reach pharmacies [8–10]. Flexibility regarding medication choice, dose titration, and treatment duration are required to increase accessibility of medications such as OAT [11]. Beyond these adaptations, new and innovative approaches are required to connect people who use illicit drugs to health care and pharmacy services.

Navigating services access and advocating for alternatives when first-line interventions are not appropriate requires time, energy, and knowledge. Peers (i.e. people with lived experience of the topic at hand, in this case illicit substance use) are effective patient navigators in health systems, not just for substance use, but for other conditions such as diabetes and heart disease. [12, 13]

In this case report, a peer-led approach being carried out in a rural community in BC is presented. While the language of “peer-led” can be used in the context of a range of programs, with diverse objectives, structures, and outcomes, in the context of this manuscript, the peer-led approach represents the work carried out by one peer (JM), to collaborate with health service providers (i.e. prescriber, pharmacist), to ensure access to and continuity of medications for patients accessing services at a community clinic. We outline what the program offers, how services are delivered, and program outcomes. The report is written from the positionality of the First Author, who is an Indigenous (Heiltsuk, Kwakuitl) woman with more than a decade of experience working in harm reduction, who brings an Urban Indigenous worldview to the manuscript and has worked collaboratively with the Second Author (JM) to document the peer-led approach. The manuscript brings attention to the intersections of this program’s peer-led approach with an Indigenous harm reduction approach [14, 15].

### Case presentation

**Fig. 1:**
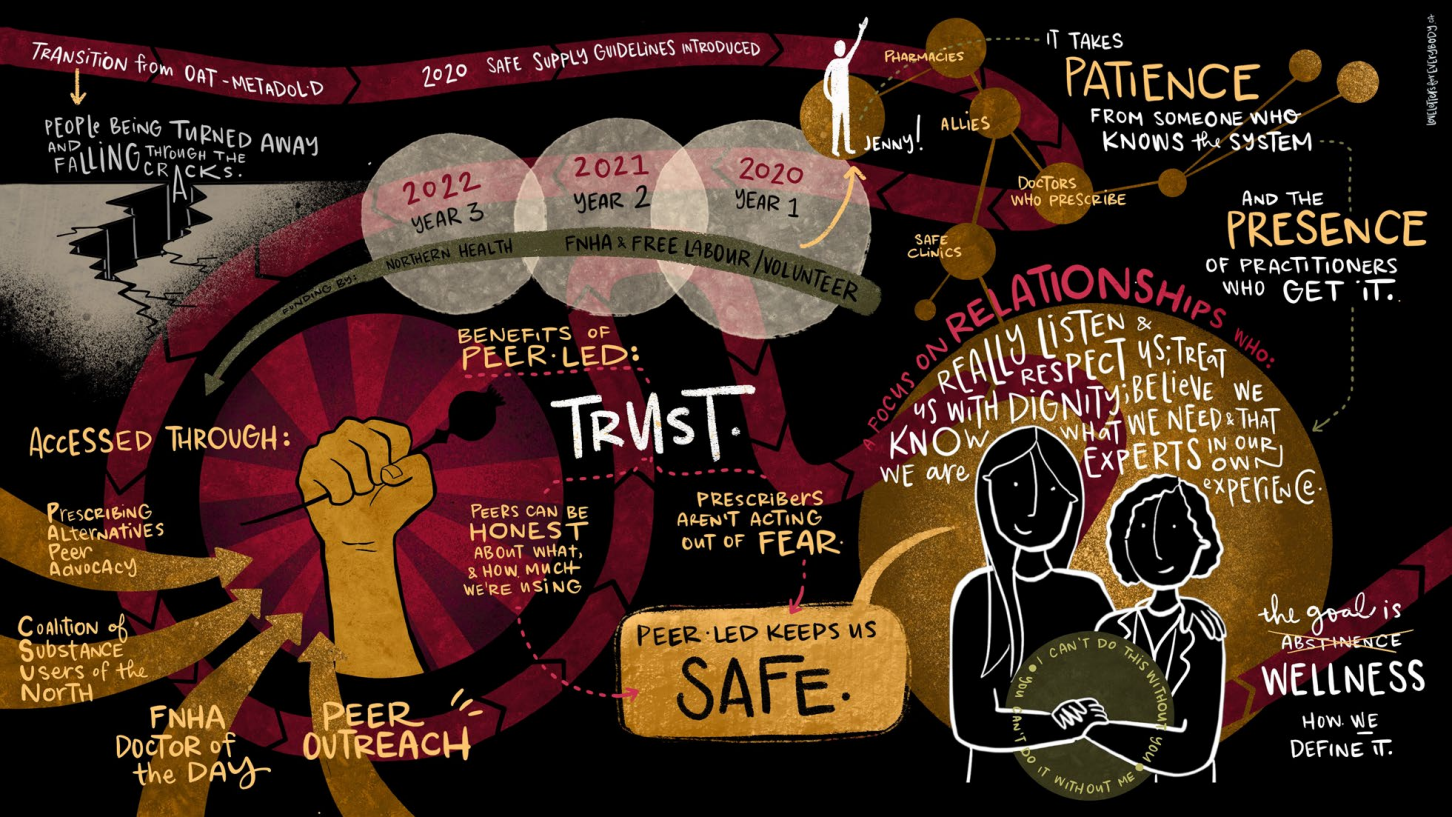
Peer-led medication delivery program in rural Northern BC

**Fig. 2:**
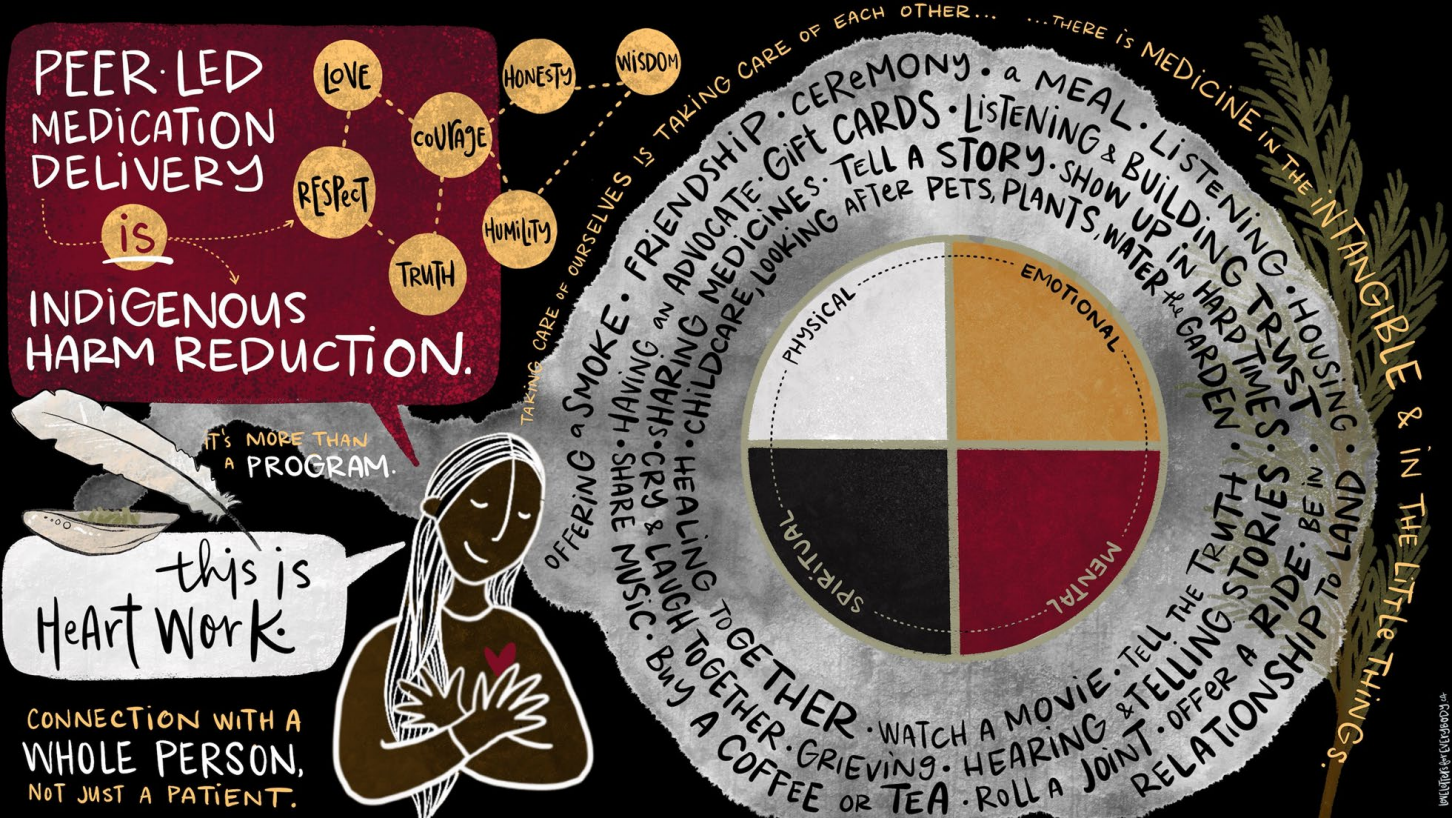
Peer-led medication delivery is Indigenous harm reduction

**Fig. 3:**
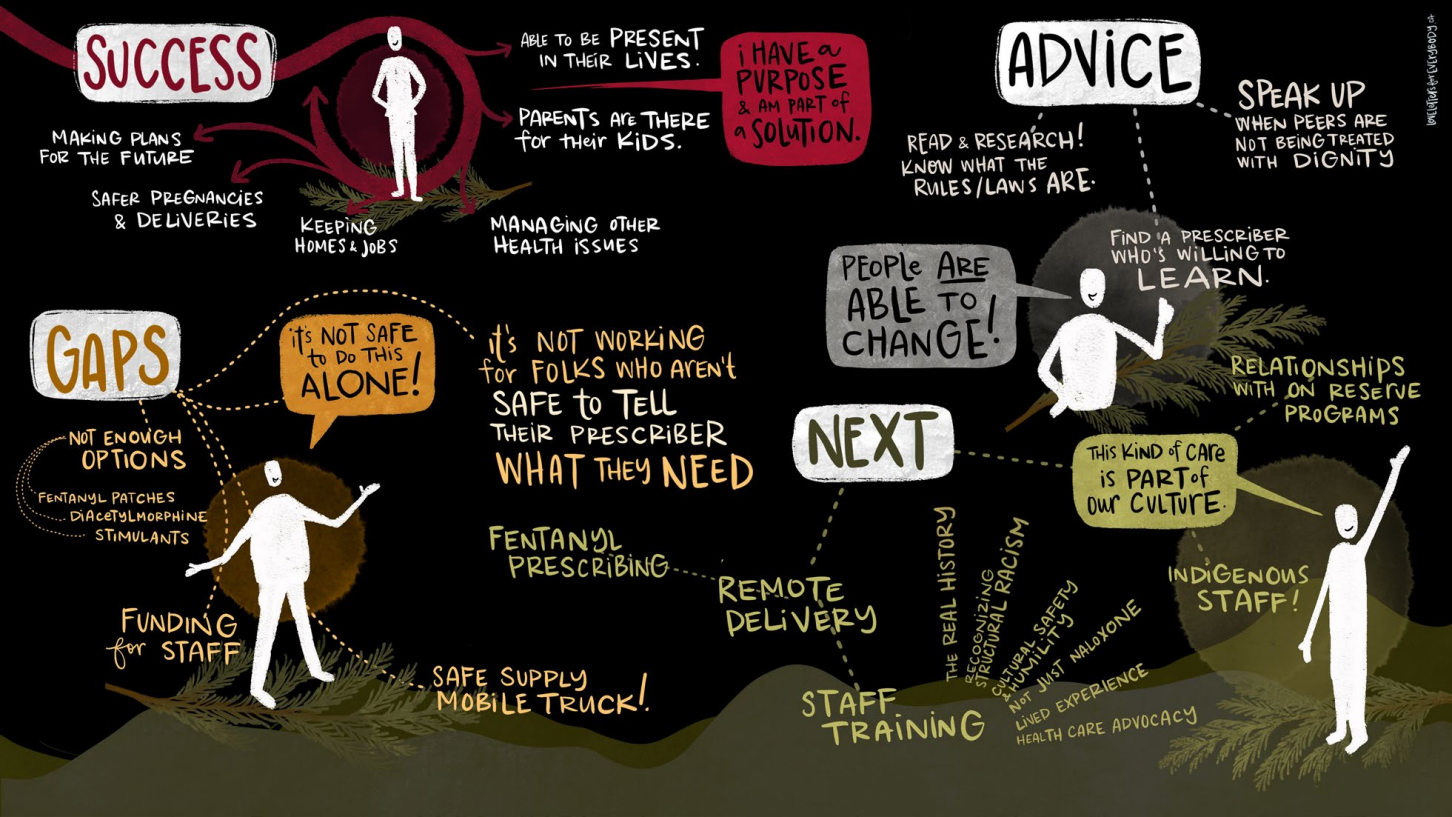
Markers of success and next steps

The case presentation is organized around three graphic illustrations which represent the experience of the peer who leads this intervention (JM), outlining what she does (Fig. 1), how she does it (Fig. 2), and program outcomes (Fig. 3).

### Delivering the service

In the present program, a peer with lived experience of entrenched homelessness, addiction, incarceration (JM) has been serving in her capacity as a peer since 2010, when she began attending the clinic in a town in rural British Columbia. Since then, she has worked as a community advocate for harm reduction, substance use services, with the Coalition of Substance Users of the North. Through this role, she began advocating for new clients to be taken into the OAT clinic, formally began engaging in the work outlined in the manuscript in March 2020, in the context of COVID-19, when a new policy was introduced in BC that provided guidance for physicians to prescribe a range of alternatives to the unregulated drug supply (See Table 1). Medications delivered by the peer include opioids (i.e. opioid agonist treatment (OAT) medications, methadone, buprenorphine, slow release oral morphine, prescribed safer supply medications, hydromorphone tablets, M-Eslon), stimulant medications (i.e. methylphenidate, dextroamphetamine). The peer initially engaged in this work as a volunteer, then was compensated with honorarium, is now compensated with a wage as a full-time employee

**Table 1:** Opioid agonist treatment and safe supply medications

Medication type	Medication name
Opioid agonist treatment	
	Methadone
	Buprenorphine/naloxone
	Extended-release buprenorphine
	Slow-release oral morphine
Opioid safe supply	
	Hydromorphone tablet
	M-eslon (morphine)
Stimulant safe supply	
	Dextroamphetamine
	Methylphenidate

Medications listed are those available for the treatment of opioid use disorder in BC^20^ and those listed in BC’s Provincial Risk Mitigation Guidance^21^

The peer carries out medication delivery by building trusting relationships with her clients. She serves as a bridge between the health care system and patients, meeting them where they are. Some clients had pre-existing relationships with the peer in community, while others who were already clinic clients built the connection with the peer at the clinic. The peer relies on existing, established relationships with health practitioners (i.e. prescribing physician, pharmacist, nurses), to navigate patient service access. She facilitates the following series of contacts for patients: initial appointment booking, reminders, rides to and from and accompaniment to medical appointments, medication pickup and delivery (to housing). Medication discontinuation is avoided by check-ins, reminders, and by regular communication between the peer on behalf of the patient, and the physician’s office and pharmacist.

Since March 2020, this program has connected patients with care, including people most vulnerable to overdose and related harms (e.g. pregnant people, and people with concurrent hepatitis C and HIV) who may have otherwise not been engaged in health services. The program is operated out of an OAT clinic. The only eligibility criteria for accessing care is current opioid use. Many clients self-identify as Indigenous however Indigenous ancestry is not a requirement to become a patient at the clinic. For patients who do not use opioids and who are Indigenous, the peer makes referrals to the FNHA virtual clinic, which serves Indigenous people in BC who are seeking substance use services.

The peer has regular contact (every 1–4 days) with approximately 50 people and navigates medication delivery and appointments for approximately 10–15 people each day. The peer supports contact with care for OAT medications and safe supply medications (See Table 1 for medication list). While the peer’s efforts focus on medications for substance use, she also supports clients other health needs, including providing attention to gender specific service needs relating to mental health [16] and parenting. For example, the peer drives pregnant clients to prenatal care visits in a neighboring community. The peer also supports medication access for related conditions known to be overrepresented among people who are accessing the unregulated drug supply, including infectious diseases [17] such as HCV, HIV, and chronic conditions such as diabetes and cardiovascular disease [18–20] to support a wholistic sense of health for each client.

Supporting clients with accessing housing is a critical component of the peer’s work, and access to medication and treatment requires first establishing access to secure and stable housing. The peer engages in a number of activities to promote connection to housing including filing “Intent to Rent” forms for clients with the local Ministry of Housing office, supporting clients to file with the Residential Tenancy Branch if they have been evicted, attending local shelters and hotels and communicating with managers to advocate for clients to access rooms.

### How (see Fig. 2): peer-led medication delivery is indigenous harm reduction

It is critical to note that this program, while focused on medication delivery, is much more than medication delivery. The peer follows an Indigenous Harm Reduction approach [14, 15], seeing each patient as a whole person and responds to their needs as a whole person. This is accomplished by relying on and practicing the principles of relationship and care, knowledge and wisdom, strength and protection, and healing. While the peer does not self-identify as Indigenous, she brings significant connection to the community, and through her lived experience and expertise in navigating community substance use services has been able to establish trusting relationships with clients and practice Indigenous harm reduction principles. Examples of how the peer practices these principles has been documented below:

The principle of relationships and care relies on establishing relationships, which are made possible by the Peer’s lived experience, which equips her with compassion, empathy and understanding. These relationships are built by connecting with the needs the person has at any particular time whether for a meal, someone to be a friend and listen, or to help with pet care. Indigenous harm reduction requires building of relationships and trust as a foundation which can then support connection to “mainstream” harm reduction services (e.g. attending an appointment with an OAT prescriber).

The principle of knowledge and wisdom relies on acknowledgement of clients’ experiences of stigma and shame, as factors that shape their interactions or lack thereof with the health system. Indigenous peoples experience systemic and internalized stigma, which is compounded among people actively using illegal substances. These layers of stigma can be broken down by interacting with someone with shared lived experience, who understands these baseline experiences as a barrier that needs to be navigated in moving toward engaging with the health system. The principle of strength and protection is practiced by the Peer, in acknowledging the critical role of culture and tradition in wellness, and the connections the peer is able to facilitate to Indigenous health service providers (e.g. Elders, and physicians working with the FNHA).

The principle of healing relies on supporting clients toward self-acceptance as part of the path to wellness and is established by approaching interactions with non-judgment, and unconditional care and support regardless of current positions (e.g. substance use, housing status, criminal legal system interaction).

### Program outcomes and next steps (see Fig. 3)

The peer described the broad range of outcomes she has witnessed in program clients including making plans for the future, safer pregnancies and deliveries, keeping homes and jobs, managing other health issues, parents being able to be around for their kids, and being more present in their lives. Despite these positive outcomes, there are several existing gaps in services that remain, including the need for a broader range of medications to meet a diversity of needs, and engage people who remain outside of the care system. Along with an expanded range of medications is the need for medication options that meet a diversity of patient preferences regarding routes of administration. Recent data demonstrate that smoking is now the most common and preferred version of opioid use in BC among people accessing harm reduction services [21], however currently available OAT and PSS medications that the peer delivers include only oral options. Furthermore, preferences will depend on motivations for use [22], and with diverse motivations for use across clients, a diversity of medications will be required to best meet client needs and promote separation from the illicit drug supply, to support reductions in overdose rates. Furthermore, polysubstance use is on the rise in BC and has been associated with elevated risk of both fatal, and non-fatal overdose [23, 24], and must be considered in discussions regarding implementation and expansion of OAT and PSS medications.

There is a need for continued training for staff in health systems around Indigenous harm reduction principles and approaches to decolonizing care [14, 15], including hiring of Indigenous staff to lead interventions for Indigenous peoples, who remain overrepresented among people who experience overdose in BC [7].

## Discussion

In this case report, a peer-led medication delivery program has been outlined. Through an Indigenous harm reduction approach, the peer was able to establish regular and ongoing contact with clients since March 2020 to support engagement with health care and continuity of medication access, including OAT, and safe supply medications.

This program was implemented in the context of an overdose crisis in BC, where the health and substance use needs of many people remain unmet by existing services. For example, oral opioid agonist treatment (first-line treatment for opioid use disorder in BC) retention has been declining over the past decade, and when people do attempt treatment with oral OAT, induction rates are low, and minimum effective doses are often not reached [25]. There are well-known barriers to access and engagement in OAT, including known stigma facing people who use drugs and stringent protocols (e.g. urine drug screens, daily dispensation, witnessed ingestion), which make access a challenge particularly in rural and remote communities [26]. In this program, the peer served as a point of contact to care, reducing fears regarding stigma and discrimination, and easing the burden of stringent OAT protocols, for example by bringing the medication to the person, rather than requiring them to travel to a clinic daily.

Following an Indigenous harm reduction approach [14, 15], the peer was focused on meeting the needs of the whole person, rather than on the medication only. This allowed for basic social determinants of health to be met, including access to housing. Access to these necessities is an important indicator of whether or not people will be in a position to maintain access health services long-term. This is well supported by the literature, whereby people experiencing homelessness face additional barriers to substance use treatment access and are at an increased risk of OAT discontinuation [27]. Due to ongoing effects of colonialism, Indigenous peoples remain overrepresented among people experiencing overdose events and deaths [7]. As such, Indigenous-led interventions that prioritize Indigenous harm reduction approaches such as the intervention outlined in this report are needed.

In terms of barriers and challenges faced, the peer outlined some resistance to her role as she first began, in particular in interactions with the pharmacy. She noted that after only a couple of months of regular communication about the program and rapport building, this resistance had disappeared. While the peer works consistently to navigate access to housing, lack of housing options means that many clients remain unhoused. This poses an ongoing challenge to medication access and engagement, for example with medications going missing or being stolen. Housing remains a critical foundation for supporting client wellness and connection to care. Accessibility of services and care for pregnant persons remains a challenge. The peer supports pregnant clients by driving them to their prenatal care appointments; however, these appointments are not available within the community. This takes significant resources from the peer (i.e. time, cost of gas, etc.) and reduces her capacity to support additional clients.

This manuscript has highlighted the value of peer-led work and the need for further investments in peer-led programs to respond to the overdose crisis. Peers have the solutions and the passion for this work and can create bridges between patients and the health system by building trusting relationships [28]. Nevertheless, this critical knowledge and expertise is often overlooked. This paper is a call to action for the recognition of the knowledge of people with lived experience of substance use in the health system. Investment into formalizing peer-led services across BC will be required to reduce overdose risk, particularly in rural settings where barriers to health services access are elevated and rates of overdose death are high [29].

## Conclusion

This peer-led intervention is a promising approach to engaging people who remain disconnected from health services and to ensure continuity of medication to reduce overdose risk. This model could be adapted to other settings with willingness of funders and clinicians to partner with peers to support patient contact with the health system, with the goal of reducing overdose risk.

## Data Availability

Data available upon request.

## Abbreviations

BC: British Columbia
HCV: Hepatitis C virus
HIV: Human immunodeficiency virus
OAT: Opioid agonist treatment
